# The Effect of the COVID-19 Pandemic on Digital Health–Seeking Behavior: Big Data Interrupted Time-Series Analysis of Google Trends

**DOI:** 10.2196/42401

**Published:** 2023-01-16

**Authors:** Robin van Kessel, Ilias Kyriopoulos, Brian Li Han Wong, Elias Mossialos

**Affiliations:** 1 LSE Health Department of Health Policy London School of Economics and Political Science London United Kingdom; 2 Department of International Health Care and Public Health Research Institute Maastricht University Maastricht Netherlands; 3 Steering Committee Digital Health Section European Public Health Association Utrecht Netherlands; 4 Institute of Global Health Innovation Imperial College London London United Kingdom

**Keywords:** digital health, healthcare seeking behaviour, big data, real-world data, data, COVID-19, pandemic, Google Trends, telehealth

## Abstract

**Background:**

Due to the emergency responses early in the COVID-19 pandemic, the use of digital health in health care increased abruptly. However, it remains unclear whether this introduction was sustained in the long term, especially with patients being able to decide between digital and traditional health services once the latter regained their functionality throughout the COVID-19 pandemic.

**Objective:**

We aim to understand how the public interest in digital health changed as proxy for digital health–seeking behavior and to what extent this change was sustainable over time.

**Methods:**

We used an interrupted time-series analysis of Google Trends data with break points on March 11, 2020 (declaration of COVID-19 as a pandemic by the World Health Organization), and December 20, 2020 (the announcement of the first COVID-19 vaccines). Nationally representative time-series data from February 2019 to August 2021 were extracted from Google Trends for 6 countries with English as their dominant language: Canada, the United States, the United Kingdom, New Zealand, Australia, and Ireland. We measured the changes in relative search volumes of the keywords *online doctor*, *telehealth*, *online health*, *telemedicine*, and *health app*. In doing so, we capture the prepandemic trend, the immediate change due to the announcement of COVID-19 being a pandemic, and the gradual change after the announcement.

**Results:**

Digital health search volumes immediately increased in all countries under study after the announcement of COVID-19 being a pandemic. There was some variation in what keywords were used per country. However, searches declined after this immediate spike, sometimes reverting to prepandemic levels. The announcement of COVID-19 vaccines did not consistently impact digital health search volumes in the countries under study. The exception is the search volume of *health app*, which was observed as either being stable or gradually increasing during the pandemic.

**Conclusions:**

Our findings suggest that the increased public interest in digital health associated with the pandemic did not sustain, alluding to remaining structural barriers. Further building of digital health capacity and developing robust digital health governance frameworks remain crucial to facilitating sustainable digital health transformation.

## Introduction

On March 11, 2020, the World Health Organization announced that COVID-19 has been classified as a pandemic, disrupting health care services worldwide. Evidence from a systematic review shows a median 42% decrease in health care visits, 28% decrease in admissions, 31% reduction in diagnostics, and 29% reduction in therapeutics, resulting in an overall 37% decrease in health care usage [1]. As a result, digital health has exploded during the early stages of the COVID-19 pandemic [2-4]. Digital health generally refers to the use of internet solutions, big data, and communications technologies to collect, share, and manage health information to improve both individual and public health, as well as to identify symptoms, plan treatment, monitor key health parameters, and monitor progress and treatment effects [5-7]. While digital health saw tremendous uptake early in the pandemic [4,8], no information exists on whether this uptake was sustainable [9].

The sudden disruption of traditional health services may have led health ecosystems to digitalize out of a need to survive, highlighted by the emergency transition to a digital paradigm and subsequent changes when the pandemic started [2]. Implementing digital health solutions is complex and relies on many institutional factors, cultural and behavioral traits, and health system characteristics [9,10]. For instance, the design process of digital health solutions is indicative of which populations it will be able to reach and, equally importantly, what population groups will experience difficulties in accessing and using the tool [3,11,12]. Policy environments are vital to laying the foundation of how conducive a health system is to adopting a digital health solution and how health professionals are trained in the field of digital health [3,11,13]. The readiness and willingness of digital health users are crucial elements in adopting digital health solutions [2,10]. A recent analysis of digital skills in the European Union—frequently regarded as a leading region in terms of digital skills—found clear discrepancies across the European region [14]. Certain population groups seem to fare more favorably in a digital world: people who are younger, higher educated, male, live in urban regions, and are either students or employed consistently report higher internet access and digital skills [14-17]. Consequently, if digital health services were to be structurally introduced now, only certain population groups could fully benefit from these services. Paradoxically, population groups that have higher needs for health care and could potentially benefit most from these innovations are the ones that would experience the highest barriers to access [8,11,12].

With traditional health services recovering, patients could decide whether they prefer to seek traditional or digital health care, which often starts with web-based search engines [18]. Although the internet cannot substitute health professionals as a sole health information source due to a combination of the prominence of misinformation and a lack of health, digital, and science literacy [16,19-21], search engine data can be instrumental in understanding the general preference of populations in exploring the possibility of using digital health [22,23]. While web-based searches can be information- or curiosity-driven, previous research has shown that web-based search behavior is strongly correlated with the actual (health care) needs of the population and forms an integral part of a pathway to actual communication with health care providers [22-25].

This study aims to understand how the public interest in digital health changed as proxy for digital health–seeking behavior in Australia, Canada, New Zealand, the United Kingdom, the United States, and Ireland when COVID-19 was declared a pandemic on March 11, 2020, and whether any observed changes were sustained over time. In particular, we hypothesize that (1) interest in digital health rose when the pandemic was announced and (2) this increased public interest also declined over time. It is important to emphasize that this study does not cover the demand but only the public interest in digital health care, which may not always translate into demand.

## Methods

### Overview

Google Trends is a principal tool used to study trends and patterns of search engine queries [22]. It is an open-access big data repository that provides real-time information on Google queries from 2004 onward [26], solving issues that arise with conventional, time-consuming survey methods [26]. These data showcase how popular specific search terms were in certain countries. The main advantage of Google Trends is that it captures the revealed, and not stated, users' preferences [26], making it possible to obtain data that would be difficult to collect otherwise. The advantage of revealed user data is that they are based on actual decisions, meaning that there is no need to assume that participants will respond to simulated situations as is the case with stated user data. As a result, revealed user data are characterized by high reliability and face validity [27]. Google Trends has shown to be a viable tool to understand, monitor, and even forecast information-seeking trends and public interest and is becoming an increasingly popular method for assessing population preferences in health research [22,28,29]. It has been applied to various health-related topics such as mental health, vaccine hesitancy, and infodemic surveillance [25,30,31].

All Google Trends data points are normalized and scaled [26], meaning that the number of searches for a specific term is divided by the total number of searches for all topics at a particular location and within the specified time frame, resulting in a normalized score. All normalized scores are scaled between 0 and 100 points, yielding relative search volumes. These relative search volumes represent search interest relative to the month with the highest search interest. Google adjusts relative search volumes for internet access and population size. We followed the established methodological guidelines for using Google Trends in infodemiology and infoveillance [26].

### Keyword, Time Frame, and Country Selection

Based on the digital health–focused search strings used in recent systematic reviews [32-34], we used 5 keywords to monitor web-based search interests: *online doctor*, *telehealth*, *online health*, *telemedicine*, and *health app*. We also explored *digital health*, *digital therapeutics*, *telecare*, *telemonitoring*, and *virtual health*, yet these terms' search volumes were negligible (<1) and were therefore excluded. By using quotation marks in our data extraction commands (eg, “online doctor”), we prevented keywords with a common term (ie, *online doctor* and *online health* or *online health* and *health app*) from including search results across multiple keywords.

We extracted the weekly relative search volume data for Canada, the United States, the United Kingdom, New Zealand, Australia, and Ireland from February 1, 2019, to August 1, 2021 (n=780 country-weeks). These countries were chosen because they share English as their dominant language, 90%-97% of their total population has access to the internet [35], and they provide a varied representation in policy landscape regarding digital health [11,13]. Google is also used for 87% to 93% of the web-based search queries in these countries [36-38], meaning that our data accurately capture the search behavior of the vast majority of the population of these countries. As such, we can reliably measure public interest in digital health during the pandemic [28].

### Empirical Approach

To assess both the short- and long-term effects of the pandemic on Google search volumes, we used a single-group interrupted time-series design [39,40], which is suitable for evaluating population-level effects of critical events (eg, interventions, policy innovations, or public health crises) that have occurred at a clearly defined point in time [41]. Our design adopts a segmented regression analysis approach that allows for the identification of 3 effects by comparing pre- and postevent trends [41,42]: (1) the pre-event slope, indicating how the outcome of interest was changing prior to the occurrence of the critical event; (2) the change in intercept, identifying the immediate change following the critical event; and (3) the postevent slope, capturing the gradual change in the period after the critical event. In this study, the critical events are the start of the pandemic on March 11, 2020, and the announcement of the vaccines on December 20, 2020. The start of the pandemic was chosen because of the shock it brought to health system functioning and the public attention it raised about the health risks associated with COVID-19 [1]. The vaccine announcement was chosen as this signaled the start of a new period where it was possible to partially return to the prepandemic status quo [43].

To account for the heterogenous spread of COVID-19 news across the studied countries, we tested the break point of March 11, 2020, using the Chow *F* test [44]. We also performed a sequential estimation using unknown break points to test for minor variations in the break point location to determine the optimal break point for this analysis [45-47].

This approach addresses internal validity constraints and represents a methodologically robust design for measuring the impact of critical events [48], and has been widely used in empirical work in the field of public health [49-51]. We also computed 7-day moving averages for active COVID-19 cases and COVID-19 deaths using Oxford Government Policy Tracker data [52]. These moving averages were added as covariates, since trends in COVID-19 cases and deaths may affect (digital) health care–seeking behavior during the pandemic. Our segmented regression models were fit using Newey-West SEs, which are designed to account for autocorrelation and potential heteroskedasticity in time-series data [53]. We conducted a postestimation analysis to capture the exact postintervention trend.

To ensure that we fit a model that accounts for the correct autocorrelation structure, we performed Cumby-Huizinga tests for autocorrelation for each individual segmented regression model [54]. The appropriate lag was determined by performing Cumby-Huizinga tests on segmented regression models without lag. Finally, we conducted a sensitivity analysis to verify the robustness of the segmented regression model, consistent with a placebo intervention using the time period between February 1, 2017, and August 1, 2019. Data were extracted using R (version 4.1.2; The R Foundation) and the analysis was performed using Stata/MP (version 17.0; StataCorp).

## Results

### Descriptive Analysis

**Figure 1:**
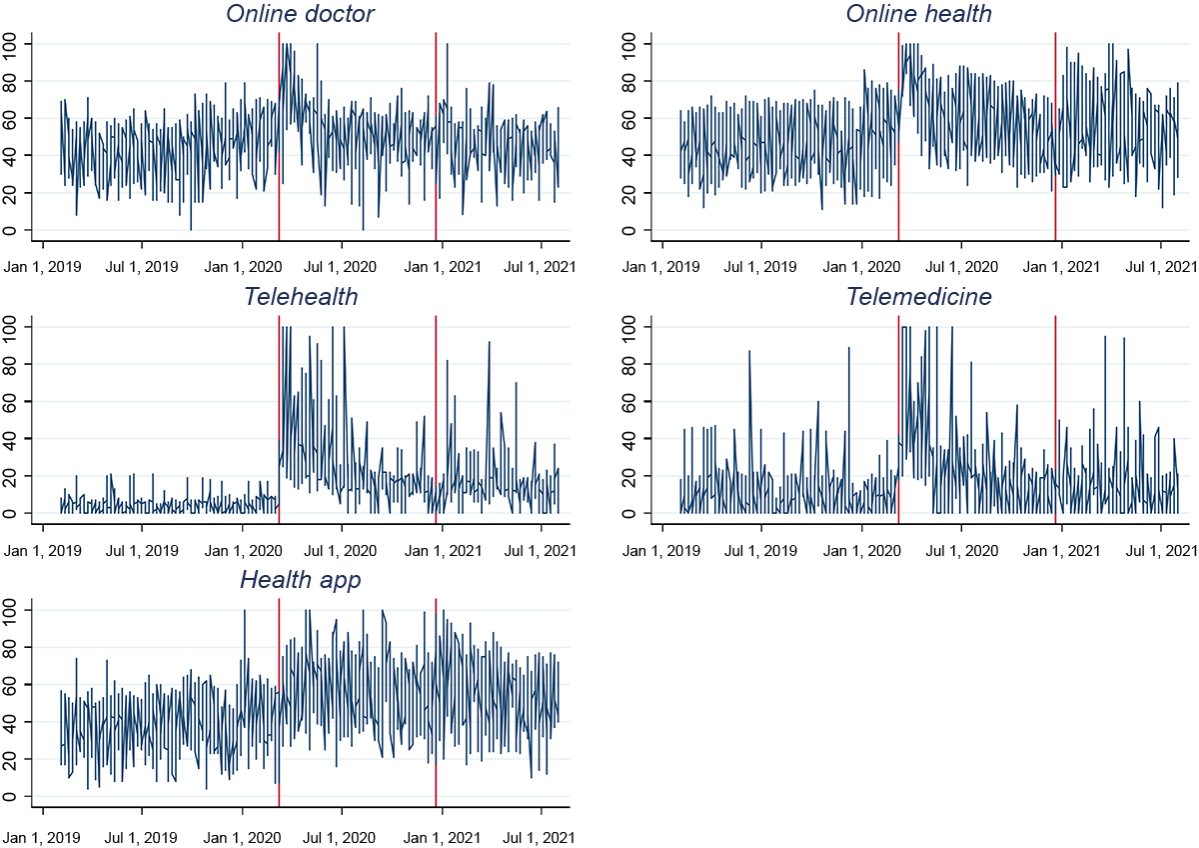
Google Trends search volumes before and after the announcement of the COVID-19 pandemic. The vertical axis shows the average search volume (scaled from 0 to 100) before and after the pandemic was announced and after the announcement of conditional market authorization of the first COVID-19 vaccines.

When comparing Google search volumes before and after the pandemic announcement on March 11, 2020 (shown in Figure 1), we observed an immediate increase in the relative search volumes of *online doctor, online health, telehealth,* and *telemedicine*. The search volume of *health app* did not indicate an immediate change, though the search volumes increased over time. We also observed a gradual reduction in the search trends of *online doctor, online health, telehealth,* and *telemedicine*, while that of *health app* remained stable. Furthermore, there is limited reason to assume “anticipation” as a relevant factor in Google search behavior, given that search volumes substantially increased only after the announcement of the pandemic. Airline traffic data remained constant until the week of March 9, 2020 [55], whereas daily Google Trends Mobility data of the countries under study remained stable until March 11, 2020 [56]. Based on these data, we inferred that changes in human behavior and mobility only emerged shortly after the pandemic declaration [57,58]. Country-specific Google search volumes are shown in Multimedia Appendix 1.

### Interrupted Time-Series Analysis

**Figure 2:**
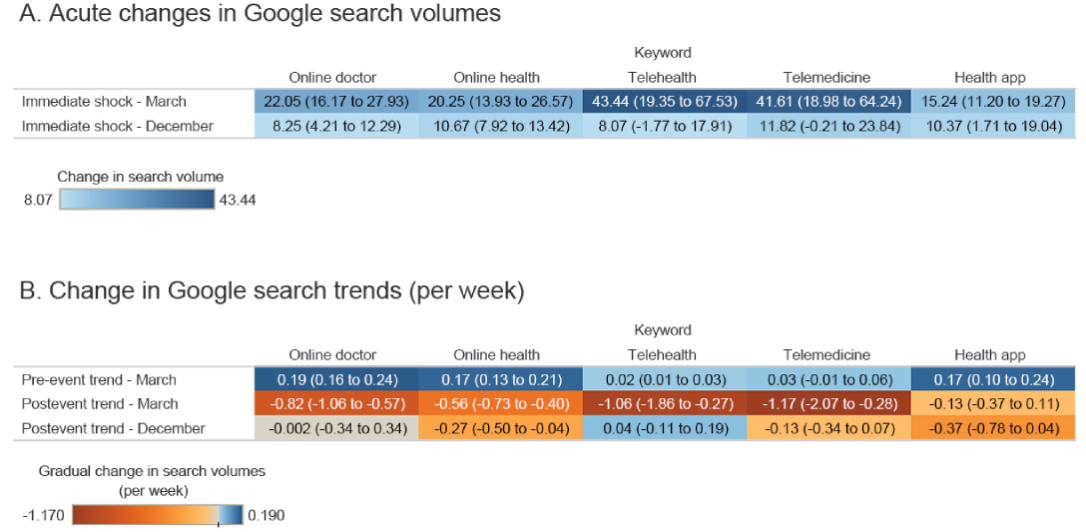
Interrupted time-series regression estimates for the relative search volumes of online doctor, online health, telehealth, telemedicine, and health app before and after the announcement of the COVID-19 pandemic and the announcement of the first COVID-19 vaccines. (A) Acute changes in the search volumes following the pandemic announcement in March 2020 and the vaccine announcement in December 2020. (B) Changes in search volumes per week before the pandemic announcement, after the pandemic announcement, and after the vaccine announcement. Values shown are estimates and 95% CIs.

When assessing all studied countries combined, we observed rising trends in the relative search volumes of *online doctor*, *online health*, *telehealth*, and *health app*. Upon the announcement of COVID-19 being a pandemic, the relative search volumes of all keywords increased significantly, though the increases in the search volumes of *telehealth* (43.44 [19.35-67.53]) and *telemedicine* (41.61 [18.98-64.24]) are particularly large. After the pandemic announcement, all keywords under study, except for *health app*, showed decreasing trends in their respective relative search volumes. After the vaccine announcement, the search volumes of *online doctor*, *online health*, and *health app* showed an acute increase. Following this event, *online health* saw a decreasing trend, while the other keywords under study revealed a stable trend. Further details are shown in Figure 2 and Table S1 in Multimedia Appendix 1.

**Figure 3:**
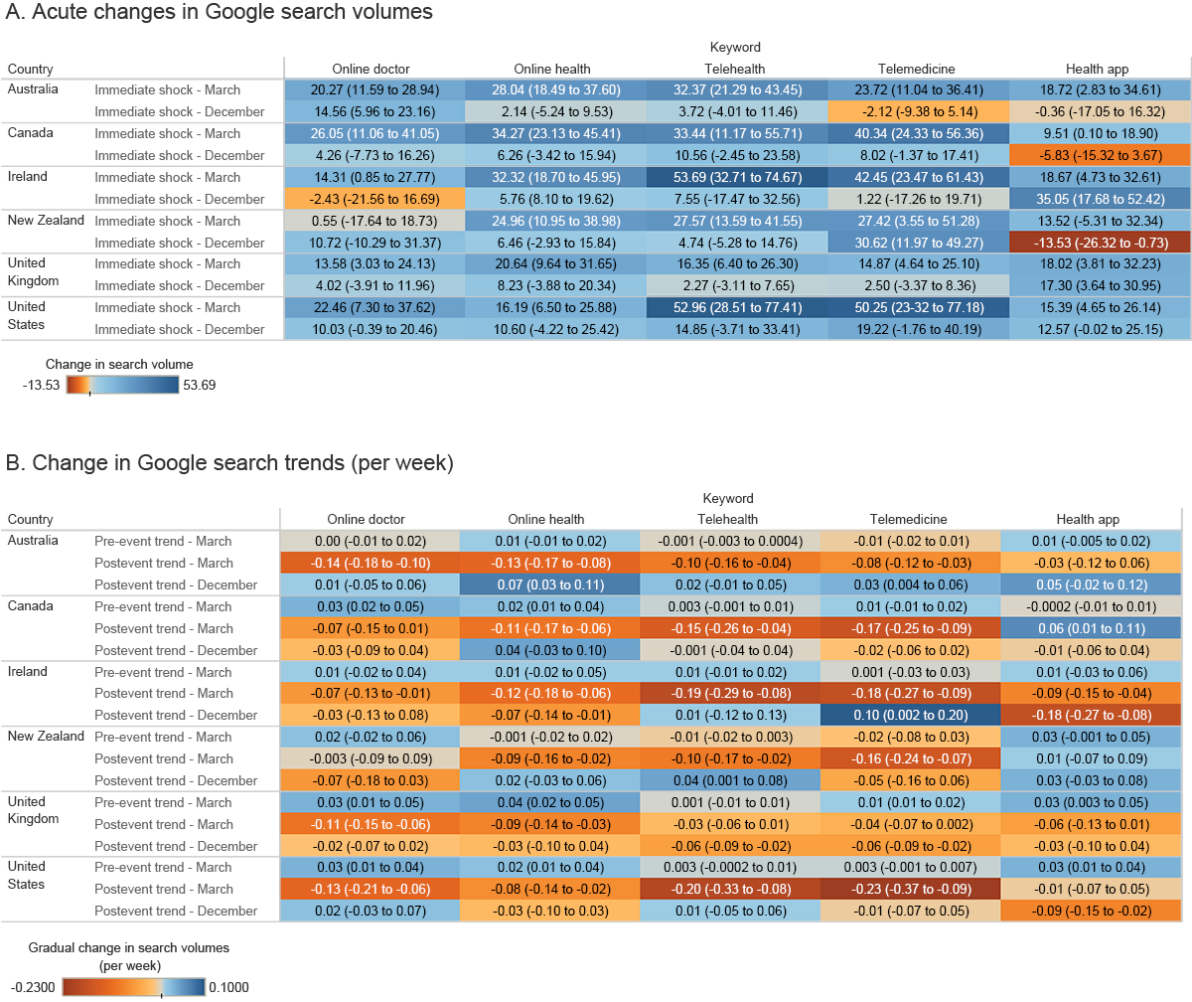
Country-specific regression estimates for the relative search volumes of online doctor, online health, telehealth, telemedicine, and health app before and after the announcement of the COVID-19 pandemic and the announcement of the first COVID-19 vaccines. (A) Acute changes in search volumes following the pandemic announcement in March 2020 and the vaccine announcement in December 2020. (B) Changes in search volumes per week before the pandemic announcement, after the pandemic announcement, and after the vaccine announcement. Values shown are estimates and 95% CIs.

Prior to the announcement of the COVID-19 pandemic, *online doctor* and *online health* showed an increase in relative search volume in Canada, the United Kingdom, and the United States, while *telemedicine* only reported an increasing search volume in the United Kingdom. The search volume of *health app* increased in the United Kingdom and the United States. Upon the announcement of the pandemic, Australia, Canada, the United Kingdom, the United States, and Ireland reported an immediate rise in the search volumes of all 5 keywords under study. In contrast, New Zealand only reported an immediate rise in *online health*, *telehealth*, and *telemedicine*. After the immediate shock, Australia and the United States reported a declining trend in the search volumes of all keywords under study except *health app*. Canada reported declining search volumes of *online health*, *telehealth*, and *telemedicine*, yet an increasing search volume in *health app*. New Zealand also reported declining search volumes of *online health*, *telehealth*, and *telemedicine*. The United Kingdom showed declining search volumes of *online doctor* and *online health*. Finally, Ireland reported a decline in all keywords under study after the immediate shock of the pandemic announcement. Further details are shown in Figure 3 and in Table S2 and Figure S7 in Multimedia Appendix 1.

Following the vaccine announcement on December 20, 2020, the search volume of *telemedicine* in New Zealand reported another immediate rise, while those of *health app* fell. Simultaneously, *health app* search volumes increased in the United Kingdom, the United States, and Ireland. After the immediate impact, Australia reported an upward trend in the search volumes of *online health* and *telemedicine*, while New Zealand reported a positive trend for the search volume of *telehealth*. In the United Kingdom, *telehealth* and *telemedicine* both reported declining trends, as well as *health app* in the United States. Finally, an increasing trend was observed in the search volume of *telemedicine* in Ireland, while *online health* and *health app* displayed a decreasing trend. Further details are also shown in Figure 2 and in Table S2 and Figure S7 in Multimedia Appendix 1.

### Robustness Checks

The break point of March 11, 2020, was tested using the Chow *F* test for the keyword *telehealth*, which resulted in a significant output (test statistic=70.82; *P*<.001), supporting the use of the week of March 8-14, 2020 as a break point. This keyword was chosen for its clearly visible break point in the descriptive analysis in all studied countries without much noise (see Figures S1-S6 in Multimedia Appendix 1). However, an assessment of the week of March 15-21, 2020, as a break point also yielded a significant result (test statistic=28.16; *P*<.001). More decisive support for our decision of the break point was obtained from the sequential estimation of unknown break points using Bai and Perron’s [45] critical values, which reported the estimated break point to be the week of March 8-14, 2020 (see Table S3 in Multimedia Appendix 1).

We used data from 2017 to 2019 and conducted placebo tests with events taking place during the same dates in 2018 (ie, March 8, 2018, and December 16, 2018) to further check the robustness of our findings. Figure S8 and Table S4 in Multimedia Appendix 1 indicate some seasonal shocks in March in *online doctor* and *online health* when using a placebo sample. However, in contrast to our main findings, the shocks in the placebo sample show a downward shock, rather than an increase, in search volume. While *online health* and *telehealth* reported a positive shock in March in Australia, the main model coefficients are significantly higher than the upper limit of the placebo coefficients' 95% CI (*online health*: 95% CI 2.19-23.80; *telehealth*: 95% CI 1.82-32.06).

## Discussion

### Principal Findings

In this study, we explored how digital health search behavior changed after the pandemic announcement and how this behavior developed in later stages of the pandemic. We observed an immediate rise in digital health search volumes in all countries under study. Simultaneously, we observed that the search volumes of the digital health–related keywords decreased after the acute increase around the World Health Organization’s pandemic announcement of March 11, 2020. These findings indicate that public interest in digital health increased as the COVID-19 pandemic was announced; yet, it also dwindled over time.

These findings are consistent with the claim that health care underwent an emergency transition to a digital paradigm when the pandemic started [2]. We also observed that after this immediate spike, digital health search volumes declined again, sometimes toward prepandemic levels. The exception to this is the search volume of *health app*, which either remained stable or gradually increased during the pandemic. This can be explained by the notion that all other keywords are meant to substitute traditional health care usage, while health apps may be used for a wider variety of purposes (eg, prevention, fitness, and managing subclinical health complaints). Additionally, a large volume of health apps was released and advocated for during the pandemic for surveillance purposes (such as recording vaccination status and generating vaccine passports), which may further contribute to explaining these findings. Seeing that vaccines were rolled out in phases across the general population, it is unsurprising that health app searches remained stable. Our placebo analysis indicated some significant seasonal trends over the time period of 2017-2019. However, these trends were almost exclusively negatively skewed. In the two cases where the trend was positive, our main finding fell outside the CI of the placebo experiment, suggesting that our main results are not driven by an artificial correlation.

It is well established that an array of factors need to align in order for a digital innovation to be adopted structurally [2,10], even more so in health care, which is characteristically one of the slowest domains in which to adopt digital innovations [59]. The beneficial effects of digital health slowly become more apparent, and our findings indicate that the general population also seeks out digital health care if traditional care is disrupted [2,11,60]. However, our findings indicate that digital health care–seeking behavior is not sustainable at the current stage of progress, seeing how the search volumes under study frequently reverted to prepandemic levels. While this may be partially explained by the return of traditional health services, it also supports previously speculated notions that key shortcomings still exist in the digital health infrastructure and competencies to fully embrace the opportunities afforded by digital health [11,12,14,61,62]. Initiatives such as the Biden Administration’s plan to provide internet access to 48 million low-income households could form an important step in removing barriers in the digital infrastructures [61], while developing educational modules and training courses available can aid in mitigating the paradoxical effects of digital health [8,11,63].

### Strengths and Limitations

This study has a number of strengths. This study is, to our knowledge, the first one that captures the prolonged effect of the COVID-19 pandemic on public interest in digital health as proxy of digital health–seeking behavior using a comprehensive list of possible search terms, which adequately captures digital health usage. While other data sets may exist, which capture digital health care usage, a unique advantage of Google Trends is the high frequency with which data are collected. This allows us to examine both short- and long-term trends from a comparative cross-country perspective. Furthermore, Google Trends data are considered revealed preference data, thus allowing for actual behaviors of Google users to be analyzed. Our countries of choice also include a mix of digitally prolific countries (eg, the United Kingdom and Australia) and more digitally hesitant countries (eg, the United States) [11,13], thus providing a balanced representation of search behavior across countries at various stages of digital health development.

This study’s limitations also need to be considered. While we were not able to ascertain sociodemographic characteristics, we need to consider the possibility of selection bias, since young people are more likely to seek health information digitally given their digital skills [14,64]. Also, population groups commonly without internet access are underrepresented in this data set (eg, people from rural communities, those from lower income brackets, lower educated people, and unemployed or retired people) [14]. Furthermore, data from only one search engine are included, and our list of keywords was not exhaustive. We attempted to test and include as many search terms as possible, yet we cannot fully predict the search terms that people use. Some linguistic nuances may have been overlooked by the systematic reviews, which underpinned our search strategy [32-34]. Additionally, our findings may not be interpreted as conclusive but should be viewed as an empirical addition to how public interest in digital health care changed at various points in the pandemic or the use of Google Trends [25,65-67]. While Google is the most popular search engine, other search engines may have also been used to seek digital health care. We did not account for population migration rates during the study period, though we do not believe that this impacted our findings due to international travel being severely restricted [68,69]. Our study does not capture digital health–seeking behavior of people who directly reached out to their general practitioner or medical contact point. Finally, our data do not allow us to capture the supply—or demand—side of digital health delivery, which may interact with the public interest for digital health services.

Recently, calls for increased and accelerated uptake of digital technologies in health care have occurred in various magnitudes [70-73]. However, our findings strongly suggest that such acceleration would be premature and could undermine the real potential of digital health delivery [15]. Vulnerable population groups, in particular, may be further disadvantaged and excluded through lack of access to digital infrastructure and underdeveloped digital skills [15,74,75]. The recently launched European Health Data Space is an example of how policy frameworks can risk exacerbating existing digital divides if policy makers do not address the underlying lack of access to digital infrastructure and underdeveloped digital skills before structurally rolling out digital health tools [12]. As such, capacity building efforts in the areas of digital infrastructure and skills should be combined with the large-scale introduction of digital health technologies to ensure that citizens and health professionals alike are in a position to recognize the value of digital health tools once they enter the health care market at large [9-11,16,63,76]. Nevertheless, the deployment of digital health tools should not cease and instead be carefully designed and targeted to specific population groups and health services because digital health is considered a type of health care modality whose value can only be derived after directly experiencing it [73]. Methodically nurturing the readiness and tension for change is, therefore, a vital element in the process of adopting digital health as a mainstream health care modality.

Policy environments are vital to the process of taking up of digital health [11,13]. National standards play an important role in creating awareness in markets, setting norms, and safeguarding basic quality dimensions of digital health [13]. So long as these factors remain underdeveloped, digital health will face substantial barriers to being implemented and assimilated into national health care settings. In fact, the absence of robust market access regulatory frameworks for digital health may also contribute to the current environment where novel tools are stuck in the proof-of-concept or pilot phase and cannot pivot to clinical adoption [3,77,78].

Finally, our findings address an overarching change in public interest for digital health, especially at the start of the pandemic, though we recognize that the development of digital health is heterogeneous and may differ among health specialties [79]. As such, more in-depth analyses of readiness to seek out digital health are warranted among health specialties. Furthermore, educational material should be developed for better acquainting civilians with the concept of digital health, building their capacity to harness the potential of digital tools and navigate digital environments [16]. More extensive educational material may be prepared for key stakeholder groups such as patients, health professionals, and policy makers [80,81]. Closer engagement with the general public and patient groups can also prove to be instrumental in developing and facilitating digital health tools that are widely accessible [11,12,61,64]. These recommendations are not only applicable in the process of adopting digital health into modern health care but also relevant to building more resilient health systems that can continue to operate in the event of a new public health crisis.

### Conclusions

In summary, this study explores how public interest in digital health changed as proxy for digital health–seeking behavior across 6 countries during the COVID-19 pandemic. We show that, after an acute increase, digital health–seeking behavior declined to levels comparable to those before the pandemic, which indicates the presence of structural problems that currently complicate the sustainable implementation of digital health. We highlight potential next steps for digital health integration in the countries under study, while acknowledging the common need for policy innovation, increasing awareness of digital health and its potential, and capacity building (including both digital infrastructure and digital literacy). While the future use of digital technologies in health is promising, we must allow both the policy landscapes and digital health literacy levels to catch up with the rapid advances in technology [2,82]. Ultimately, it is evident that more than a pandemic is needed to sustainably implement digital health.

## Appendix

Multimedia Appendix 1 Country-specific Google search volumes.

## Data Availability

Data are publicly available from the Google Trends web page. The data extraction, cleaning, and analysis code are openly available on GitHub [83].
