# Supplementary material for: The Effect of the COVID-19 Pandemic on Digital Health–Seeking Behavior: Big Data Interrupted Time-Series Analysis of Google Trends
Source: J Med Internet Res. 2023 Jan 16;25:e42401. doi: 10.2196/42401 (PMC9848442; doi:10.2196/42401)
Supplement: Multimedia Appendix 1 [file jmir_v25i1e42401_app1.docx]

**Supplementary Material**

van Kessel, R., Kyriopoulos, I., Wong, B.L.H., Mossialos, E. Has the pandemic enhanced and sustained digital health seeking behaviour? An interrupted time-series analysis of Google Trends data.

Table of Contents

[Figure S1. Google Trends search volumes in Australia before and after the announcement of the COVID-19 pandemic. 3](#_Toc118450178)

[Figure S2. Google Trends search volumes in Canada before and after the announcement of the COVID-19 pandemic. 3](#_Toc118450179)

[Figure S3. Google Trends search volumes in New Zealand before and after the announcement of the COVID-19 pandemic. 4](#_Toc118450180)

[Figure S4. Google Trends search volumes in the United Kingdom before and after the announcement of the COVID-19 pandemic. 4](#_Toc118450181)

[Figure S5. Google Trends search volumes in the United States before and after the announcement of the COVID-19 pandemic. 5](#_Toc118450182)

[Figure S6. Google Trends search volumes in Ireland before and after the announcement of the COVID-19 pandemic. 5](#_Toc118450183)

[Table S1. Regression estimates for the relative search volumes of *online doctor*, *online health*, *telehealth*, *telemedicine*, and *health app* before and after the pandemic and the vaccine announcement. 6](#_Toc118450184)

[Table S2. Country-specific estimates for the relative search volumes of *online doctor*, *online health*, *telehealth*, *telemedicine*, and *health app* before and after the pandemic and the vaccine announcement. 7](#_Toc118450185)

[Figure S7. Interrupted time-series regression analysis for the relative search volumes of online doctor, online health, telehealth, telemedicine, and health app before and after the announcement of the COVID-19 pandemic and the announcement of the first COVID-19 vaccines. 8](#_Toc118450186)

[Table S3. Results of the sequential estimation of break dates. 9](#_Toc118450187)

[Figure S8. Interrupted time-series regression analysis for the relative search volumes of online doctor, online health, telehealth, telemedicine, and health app in the time period February 2017 to August 2019. 9](#_Toc118450188)

[Table S3. Country-specific regression estimates for the relative search volumes of *online doctor*, *online health*, *telehealth*, *telemedicine*, and *health app* in the placebo period of 2017-2019. 11](#_Toc118450189)

# Figure S1. Google Trends search volumes in Australia before and after the announcement of the COVID-19 pandemic.


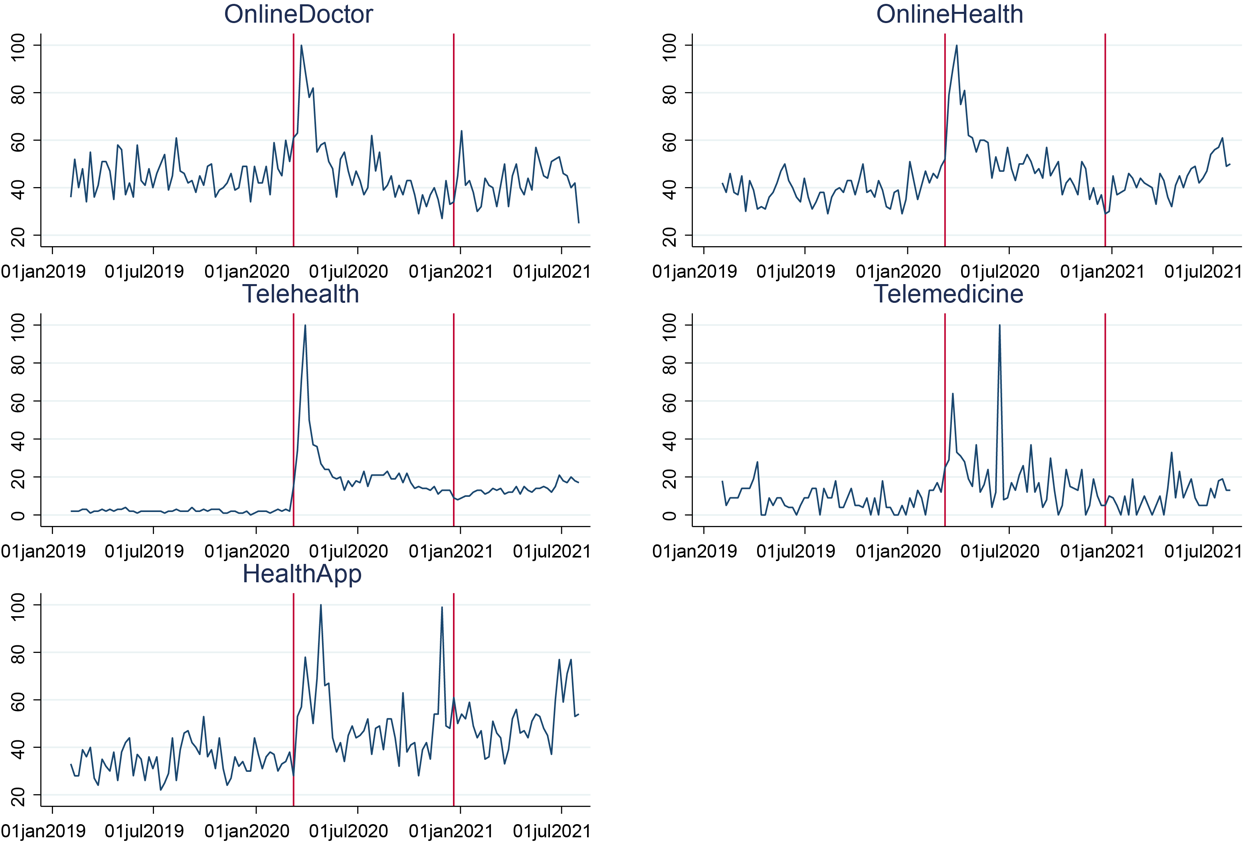


# Figure S2. Google Trends search volumes in Canada before and after the announcement of the COVID-19 pandemic.


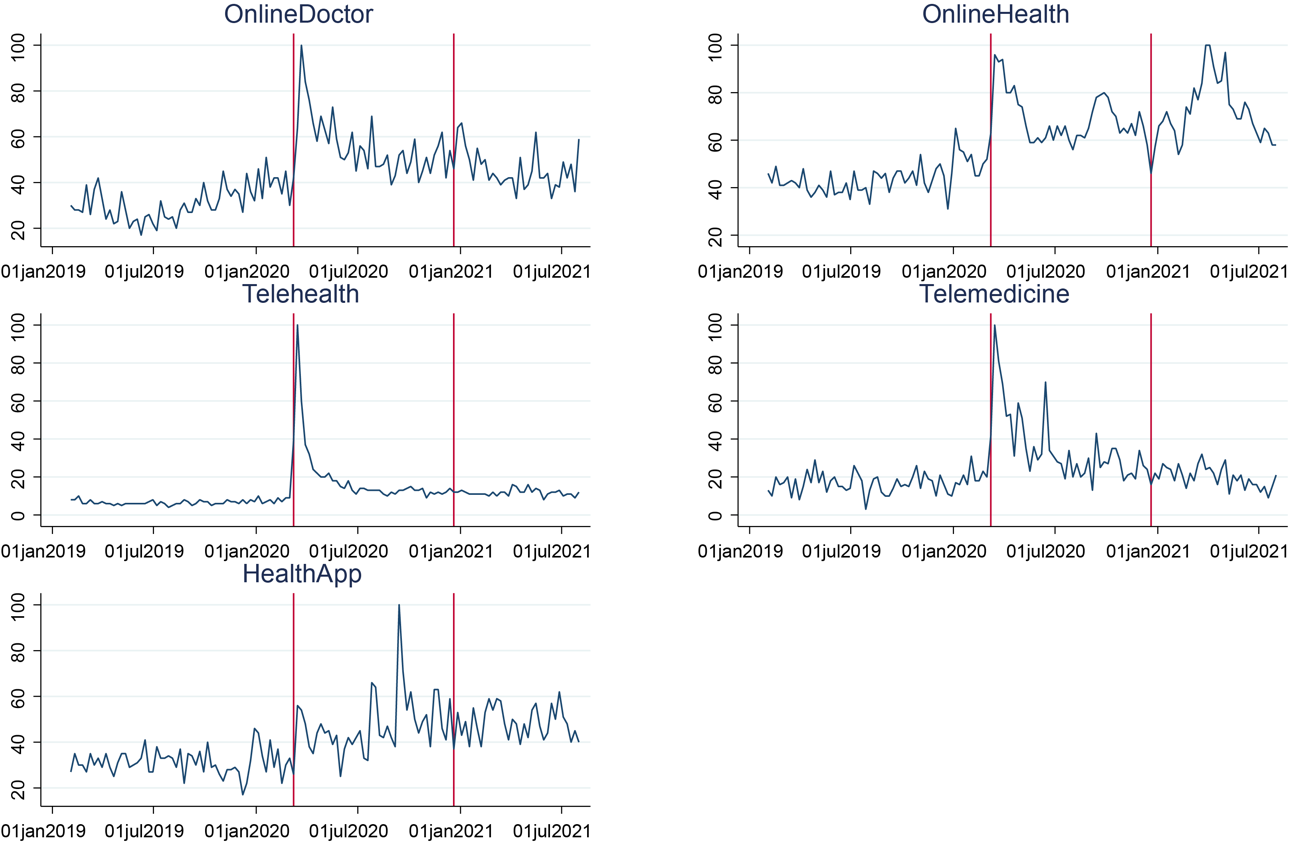


# Figure S3. Google Trends search volumes in New Zealand before and after the announcement of the COVID-19 pandemic.


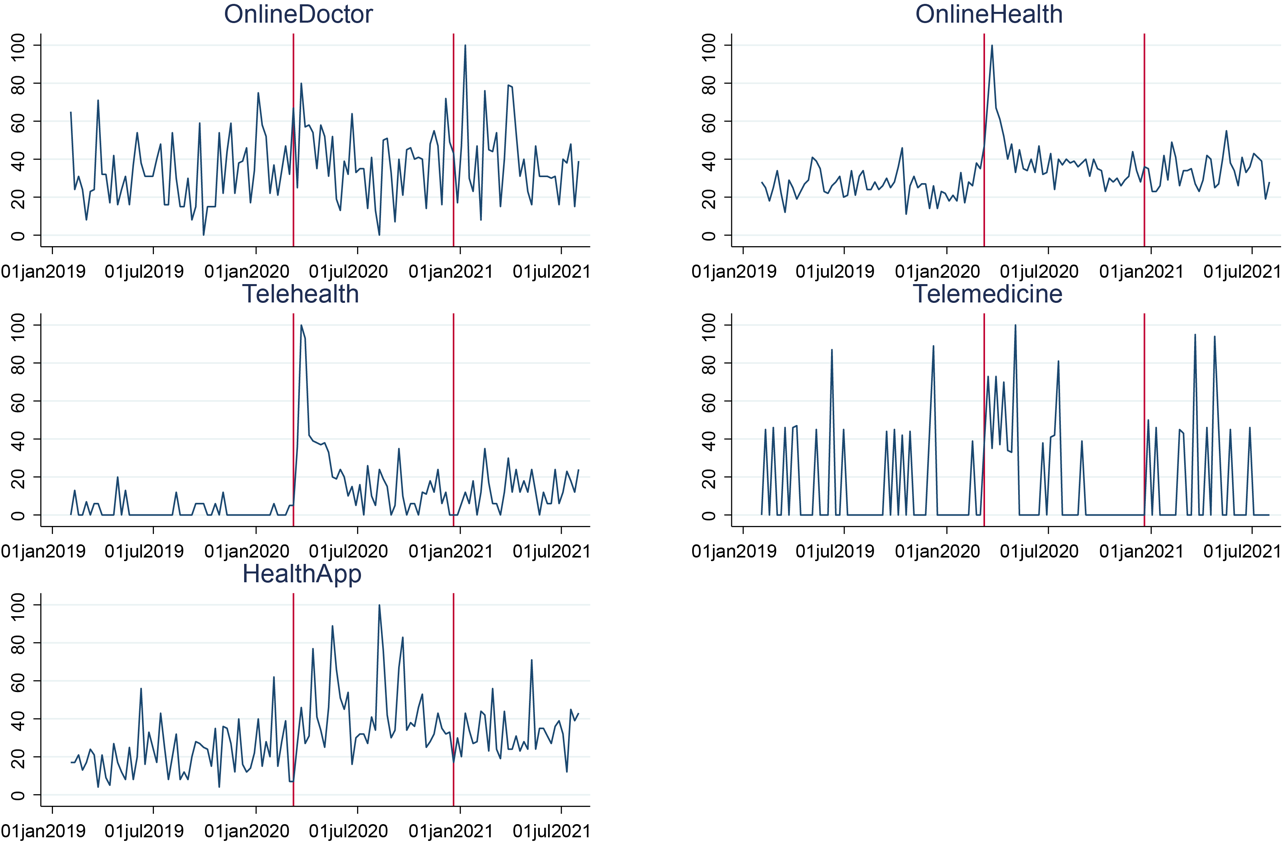


# Figure S4. Google Trends search volumes in the United Kingdom before and after the announcement of the COVID-19 pandemic.


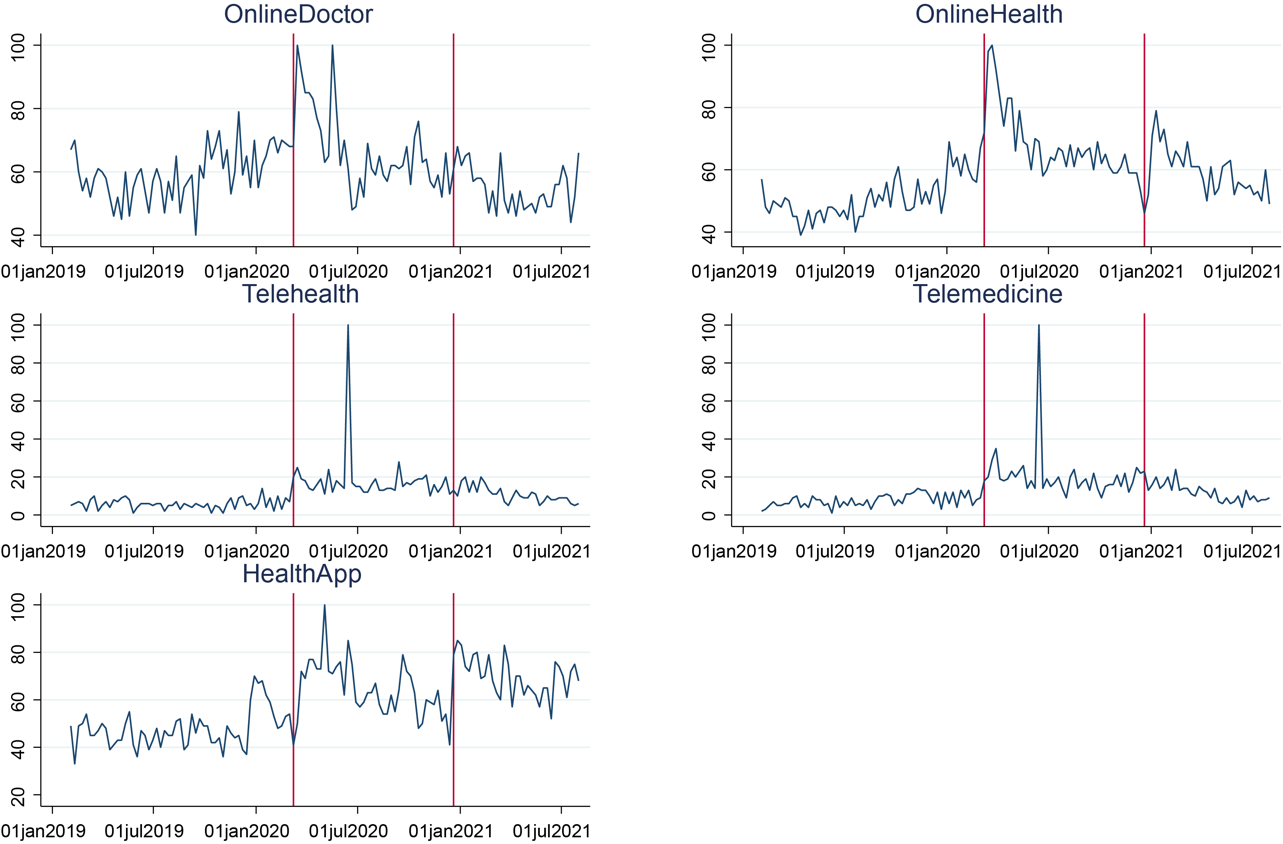


# Figure S5. Google Trends search volumes in the United States before and after the announcement of the COVID-19 pandemic.


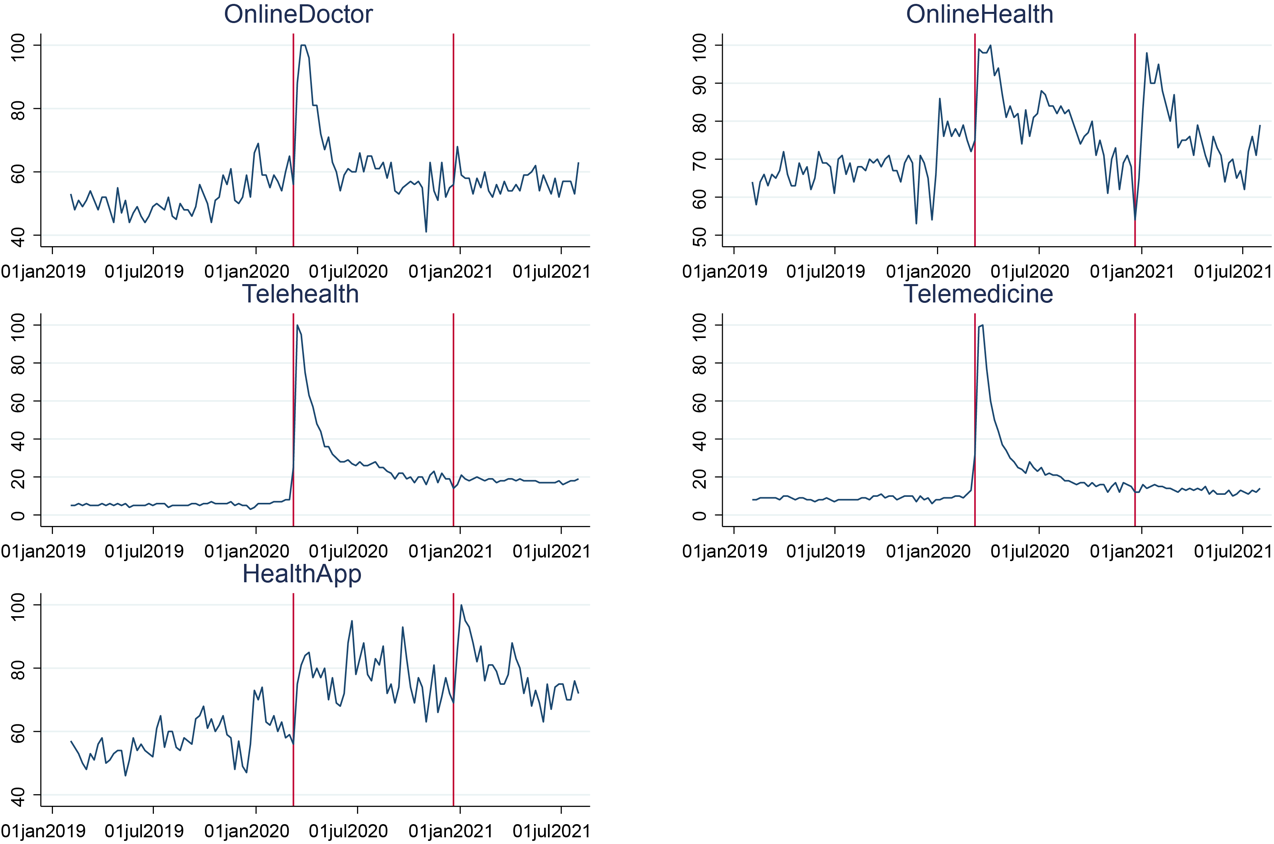


# Figure S6. Google Trends search volumes in Ireland before and after the announcement of the COVID-19 pandemic.


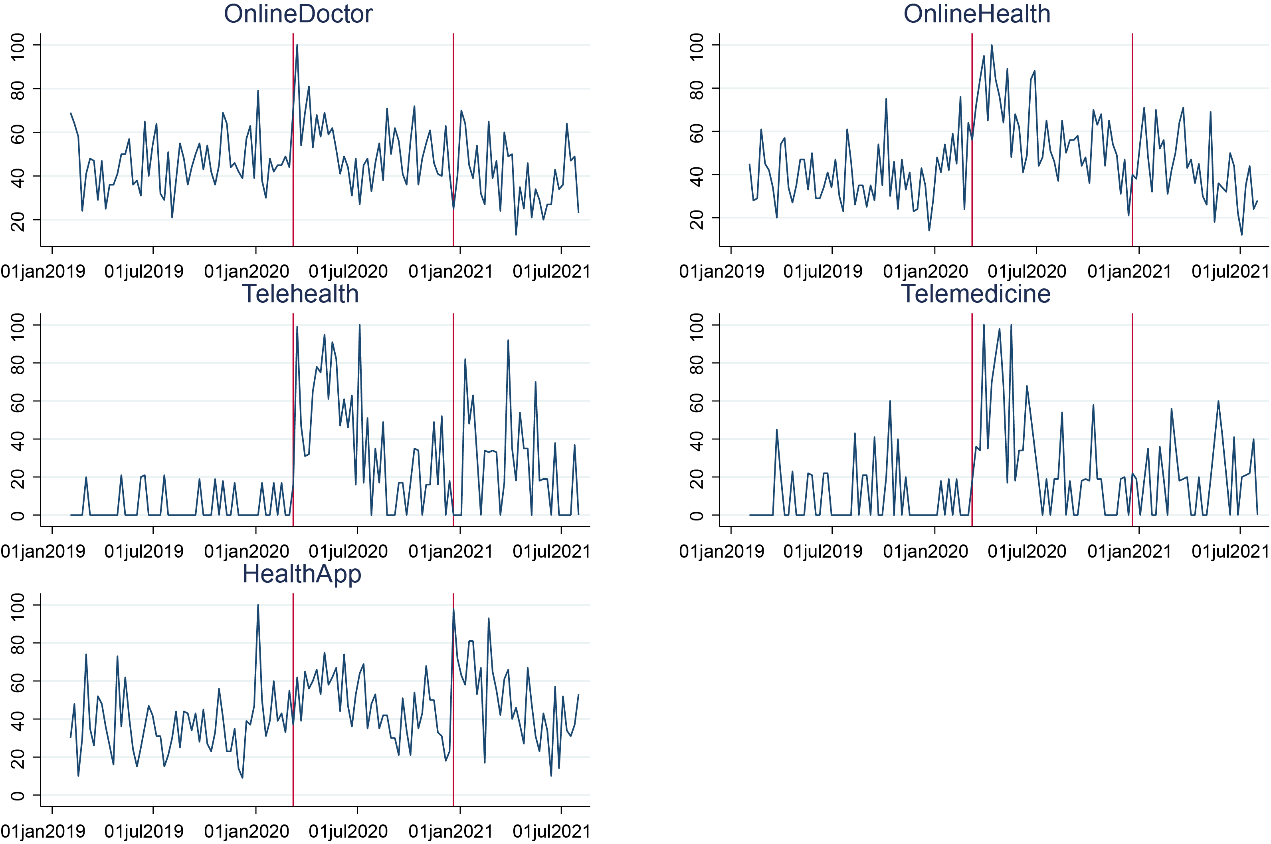


# Table S1. Regression estimates for the relative search volumes of *online doctor*, *online health*, *telehealth*, *telemedicine*, and *health app* before and after the pandemic and the vaccine announcement.

|  |  | **Coefficient** | **95% CI** | **p-value** |
| --- | --- | --- | --- | --- |
| **Online Doctor** | **Intercept** | 45.13 | 42.60 - 47.66 | < 0.001 |
|  | **Pre-event trend - March** | 0.19 | 0.16 - 0.24 | < 0.001 |
|  | **Immediate shock - March** | 22.05 | 16.17 - 27.93 | < 0.001 |
|  | **Post-event trend - March** | -0.82 | -1.06 - -0.57 | < 0.001 |
|  | **Immediate shock - December** | 8.25 | 4.21 - 12.29 | < 0.01 |
|  | **Post-event trend - December** | -0.002 | -0.34 - 0.34 | 0.99 |
| **Online Health** | **Intercept** | 56.51 | 52.91 - 60.11 | < 0.001 |
|  | **Pre-event trend - March** | 0.17 | 0.13 - 0.21 | <0.01 |
|  | **Immediate shock - March** | 20.25 | 13.93 - 26.57 | < 0.001 |
|  | **Post-event trend - March** | -0.56 | -0.73 - -0.40 | < 0.001 |
|  | **Immediate shock - December** | 10.67 | 7.92 - 13.42 | < 0.001 |
|  | **Post-event trend - December** | -0.27 | -0.50 - -0.04 | 0.03 |
| **Telehealth** | **Intercept** | 4.95 | 1.75 - 8.15 | 0.01 |
|  | **Pre-event trend - March** | 0.02 | 0.01 - 0.03 | < 0.01 |
|  | **Immediate shock - March** | 43.44 | 19.35 - 67.53 | < 0.01 |
|  | **Post-event trend - March** | -1.06 | -1.86 - -0.27 | 0.02 |
|  | **Immediate shock - December** | 8.07 | -1.77 - 17.91 | 0.09 |
|  | **Post-event trend - December** | 0.04 | -0.11 - 0.19 | 0.5 |
| **Telemedicine** | **Intercept** | 8.56 | 7.47 - 9.64 | < 0.001 |
|  | **Pre-event trend - March** | 0.03 | -0.01 - 0.06 | 0.1 |
|  | **Immediate shock - March** | 41.61 | 18.98 - 64.24 | < 0.01 |
|  | **Post-event trend - March** | -1.17 | -2.07 - -0.28 | 0.02 |
|  | **Immediate shock - December** | 11.82 | -0.21 - 23.84 | 0.05 |
|  | **Post-event trend - December** | -0.13 | -0.34 - 0.07 | 0.16 |
| **Health App** | **Intercept** | 47.17 | 44.69 - 49.65 | < 0.001 |
|  | **Pre-event trend - March** | 0.17 | 0.10 - 0.24 | < 0.01 |
|  | **Immediate shock - March** | 15.24 | 11.20 - 19.27 | < 0.001 |
|  | **Post-event trend - March** | -0.13 | -0.37 - 0.11 | 0.22 |
|  | **Immediate shock - December** | 10.37 | 1.71 - 19.04 | 0.03 |
|  | **Post-event trend - December** | -0.37 | -0.78 - 0.04 | 0.07 |

Note: Each model includes seven-day moving averages of reported COVID-19 cases and deaths as covariates.

# Table S2. Country-specific estimates for the relative search volumes of *online doctor*, *online health*, *telehealth*, *telemedicine*, and *health app* before and after the pandemic and the vaccine announcement.

|  |  | **Online Doctor** | | | **Online Health** | | | **Telehealth** | | | **Telemedicine** | | | **Health App** | | |
| --- | --- | --- | --- | --- | --- | --- | --- | --- | --- | --- | --- | --- | --- | --- | --- | --- |
|  |  | **Coefficient** | **95% CI** | **p-value** | **Coefficient** | **95% CI** | **p-value** | **Coefficient** | **95% CI** | **p-value** | **Coefficient** | **95% CI** | **p-value** | **Coefficient** | **95% CI** | **p-value** |
| **Australia** | **Intercept** | 44.34 | 40.22 - 48.46 | < 0.001 | 37.60 | 34.22 - 40.97 | < 0.001 | 2.38 | 2.01 - 2.76 | < 0.001 | 10.04 | 6.57 - 13.51 | < 0.001 | 33.17 | 30.13 - 36.21 | < 0.001 |
|  | **Pre-event trend - March** | 0.00 | -0.01 - 0.02 | 0.62 | 0.01 | -0.01 - 0.02 | 0.28 | -0.001 | -0.003 - 0.0004 | 0.13 | -0.01 | -0.02 - 0.01 | 0.22 | 0.01 | -0.005 - 0.02 | 0.24 |
|  | **Immediate shock - March** | 20.27 | 11.59 - 28.94 | < 0.001 | 28.04 | 18.49 - 37.60 | < 0.001 | 32.37 | 21.29 - 43.45 | < 0.001 | 23.72 | 11.04 - 36.41 | < 0.001 | 18.72 | 2.83 - 34.61 | 0.02 |
|  | **Post-event trend - March** | -0.14 | -0.18 - -0.10 | < 0.001 | -0.13 | -0.17 - -0.08 | < 0.001 | -0.10 | -0.16 - -0.04 | < 0.01 | -0.08 | -0.12 - -0.03 | < 0.01 | -0.03 | -0.12 - 0.06 | 0.47 |
|  | **Immediate shock - December** | 14.56 | 5.96 - 23.16 | < 0.001 | 2.14 | -5.24 - 9.53 | 0.56 | 3.72 | -4.01 - 11.46 | 0.34 | -2.12 | -9.38 - 5.14 | 0.56 | -0.36 | -17.05 - 16.32 | 0.97 |
|  | **Post-event trend - December** | 0.01 | -0.05 - 0.06 | 0.79 | 0.07 | 0.03 - 0.11 | < 0.001 | 0.02 | -0.01 - 0.05 | 0.24 | 0.03 | 0.004 - 0.06 | 0.03 | 0.05 | -0.02 - 0.12 | 0.14 |
| **Canada** | **Intercept** | 24.65 | 20.21 - 29.10 | < 0.001 | 39.00 | 35.81 - 42.19 | < 0.001 | 6.15 | 5.10 - 7.19 | < 0.001 | 15.64 | 12.91 - 18.37 | < 0.001 | 31.26 | 29.12 - 33.40 | < 0.001 |
|  | **Pre-event trend - March** | 0.03 | 0.02 - 0.05 | < 0.001 | 0.02 | 0.01 - 0.04 | < 0.01 | 0.003 | -0.001 - 0.01 | 0.19 | 0.01 | -0.01 - 0.02 | 0.27 | -0.0002 | -0.01 - 0.01 | 0.97 |
|  | **Immediate shock - March** | 26.05 | 11.06 - 41.05 | < 0.01 | 34.27 | 23.13 - 45.41 | < 0.001 | 33.44 | 11.17 - 55.71 | < 0.01 | 40.34 | 24.33 - 56.36 | < 0.001 | 9.51 | 0.10 - 18.90 | 0.05 |
|  | **Post-event trend - March** | -0.07 | -0.15 - 0.01 | 0.07 | -0.11 | -0.17 - -0.06 | < 0.001 | -0.15 | -0.26 - -0.04 | 0.01 | -0.17 | -0.25 - -0.09 | < 0.001 | 0.06 | 0.01 - 0.11 | 0.02 |
|  | **Immediate shock - December** | 4.26 | -7.73 - 16.26 | 0.48 | 6.26 | -3.42 - 15.94 | 0.20 | 10.56 | -2.45 - 23.58 | 0.11 | 8.02 | -1.37 - 17.41 | 0.09 | -5.83 | -15.32 - 3.67 | 0.23 |
|  | **Post-event trend - December** | -0.03 | -0.09 - 0.04 | 0.47 | 0.04 | -0.03 - 0.10 | 0.24 | -0.001 | -0.04 - 0.04 | 0.97 | -0.02 | -0.06 - 0.02 | 0.26 | -0.01 | -0.06 - 0.04 | 0.57 |
| **New Zealand** | **Intercept** | 28.90 | 20.06 - 37.74 | < 0.001 | 26.53 | 22.55 - 30.52 | < 0.001 | 3.51 | 0.77 - 6.25 | 0.01 | 18.11 | 5.79 - 30.42 | < 0.001 | 16.38 | 11.10 - 21.66 | < 0.001 |
|  | **Pre-event trend - March** | 0.02 | -0.02 - 0.06 | 0.37 | -0.001 | -0.02 - 0.02 | 0.85 | -0.01 | -0.02 - 0.003 | 0.18 | -0.02 | -0.08 - 0.03 | 0.34 | 0.03 | -0.001 - 0.05 | 0.06 |
|  | **Immediate shock - March** | 0.55 | -17.64 - 18.73 | 0.95 | 24.96 | 10.95 - 38.98 | < 0.01 | 27.57 | 13.59 - 41.55 | < 0.001 | 27.42 | 3.55 - 51.28 | 0.03 | 13.52 | -5.31 - 32.34 | 0.16 |
|  | **Post-event trend - March** | -0.003 | -0.09 - 0.09 | 0.94 | -0.09 | -0.16 - -0.02 | 0.01 | -0.10 | -0.17 - -0.02 | 0.01 | -0.16 | -0.24 - -0.07 | < 0.01 | 0.01 | -0.07 - 0.09 | 0.82 |
|  | **Immediate shock - December** | 10.72 | -10.29 - 31.37 | 0.31 | 6.46 | -2.93 - 15.84 | 0.18 | 4.74 | -5.28 - 14.76 | 0.35 | 30.62 | 11.97 - 49.27 | < 0.01 | -13.53 | -26.32 - -0.73 | 0.04 |
|  | **Post-event trend - December** | -0.07 | -0.18 - 0.03 | 0.18 | 0.02 | -0.03 - 0.06 | 0.54 | 0.04 | 0.001 - 0.08 | 0.05 | -0.05 | -0.16 - 0.06 | 0.36 | 0.03 | -0.03 - 0.08 | 0.32 |
| **United Kingdom** | **Intercept** | 53.45 | 48.62 - 58.29 | < 0.001 | 43.64 | 40.21 - 47.07 | < 0.001 | 5.52 | 4.20 - 6.84 | < 0.001 | 5.04 | 3.78 - 6.30 | < 0.001 | 42.68 | 39.00 - 46.35 | < 0.001 |
|  | **Pre-event trend - March** | 0.03 | 0.01 - 0.05 | < 0.01 | 0.04 | 0.02 - 0.05 | < 0.001 | 0.001 | -0.01 - 0.01 | 0.73 | 0.01 | 0.01 - 0.02 | < 0.001 | 0.03 | 0.003 - 0.05 | 0.03 |
|  | **Immediate shock - March** | 13.58 | 3.03 - 24.13 | 0.01 | 20.64 | 9.64 - 31.65 | < 0.001 | 16.35 | 6.40 - 26.30 | < 0.01 | 14.87 | 4.64 - 25.10 | < 0.01 | 18.02 | 3.81 - 32.23 | 0.01 |
|  | **Post-event trend - March** | -0.11 | -0.15 - -0.06 | <0.001 | -0.09 | -0.14 - -0.03 | < 0.01 | -0.03 | -0.06 - 0.01 | 0.15 | -0.04 | -0.07 - 0.002 | 0.07 | -0.06 | -0.13 - 0.01 | 0.09 |
|  | **Immediate shock - December** | 4.02 | -3.91 - 11.96 | 0.32 | 8.23 | -3.88 - 20.34 | 0.18 | 2.27 | -3.11 - 7.65 | 0.41 | 2.50 | -3.37 - 8.36 | 0.40 | 17.30 | 3.64 - 30.95 | 0.01 |
|  | **Post-event trend - December** | -0.02 | -0.07 - 0.02 | 0.32 | -0.03 | -0.10 - 0.04 | 0.34 | -0.06 | -0.09 - -0.02 | < 0.01 | -0.06 | -0.09 - -0.02 | < 0.01 | -0.03 | -0.10 - 0.04 | 0.39 |
| **United States** | **Intercept** | 46.31 | 43.36 - 49.24 | 0.001 | 63.55 | 61.50 - 65.61 | < 0.001 | 4.98 | 4.51 - 5.44 | < 0.001 | 8.26 | 7.57 - 8.95 | < 0.001 | 51.86 | 49.53 - 54.18 | < 0.001 |
|  | **Pre-event trend - March** | 0.03 | 0.01 - 0.04 | < 0.001 | 0.02 | 0.01 - 0.04 | < 0.01 | 0.003 | -0.0002 - 0.01 | 0.06 | 0.003 | -0.001 - 0.007 | 0.13 | 0.03 | 0.01 - 0.04 | < 0.001 |
|  | **Immediate shock - March** | 22.46 | 7.30 - 37.62 | < 0.01 | 16.19 | 6.50 - 25.88 | < 0.01 | 52.96 | 28.51 - 77.41 | < 0.001 | 50.25 | 23-32 - 77.18 | < 0.001 | 15.39 | 4.65 - 26.14 | < 0.01 |
|  | **Post-event trend - March** | -0.13 | -0.21 - -0.06 | < 0.01 | -0.08 | -0.14 - -0.02 | < 0.01 | -0.20 | -0.33 - -0.08 | < 0.01 | -0.23 | -0.37 - -0.09 | < 0.01 | -0.01 | -0.07 - 0.05 | 0.69 |
|  | **Immediate shock - December** | 10.03 | -0.39 - 20.46 | 0.06 | 10.60 | -4.22 - 25.42 | 0.16 | 14.85 | -3.71 - 33.41 | 0.12 | 19.22 | -1.76 - 40.19 | 0.07 | 12.57 | -0.02 - 25.15 | 0.05 |
|  | **Post-event trend - December** | 0.02 | -0.03 - 0.07 | 0.38 | -0.03 | -0.10 - 0.03 | 0.35 | 0.01 | -0.05 - 0.06 | 0.79 | -0.01 | -0.07 - 0.05 | 0.76 | -0.09 | -0.15 - -0.02 | 0.01 |
| **Ireland** | **Intercept** | 44.09 | 36.90 - 51.27 | < 0.001 | 37.08 | 30.43 - 43.74 | < 0.001 | 2.82 | -1.03 - 6.67 | 0.15 | 8.62 | 1.95 - 15.29 | 0.01 | 34.37 | 25.18 - 43.57 | < 0.001 |
|  | **Pre-event trend - March** | 0.01 | -0.02 - 0.04 | 0.56 | 0.01 | -0.02 - 0.05 | 0.48 | 0.01 | -0.01 - 0.02 | 0.49 | 0.001 | -0.03 - 0.03 | 0.97 | 0.01 | -0.03 - 0.06 | 0.49 |
|  | **Immediate shock - March** | 14.31 | 0.85 - 27.77 | 0.04 | 32.32 | 18.70 - 45.95 | < 0.001 | 53.69 | 32.71 - 74.67 | < 0.001 | 42.45 | 23.47 - 61.43 | < 0.001 | 18.67 | 4.73 - 32.61 | 0.01 |
|  | **Post-event trend - March** | -0.07 | -0.13 - -0.01 | 0.02 | -0.12 | -0.18 - -0.06 | < 0.001 | -0.19 | -0.29 - -0.08 | < 0.01 | -0.18 | -0.27 - -0.09 | < 0.001 | -0.09 | -0.15 - -0.04 | < 0.01 |
|  | **Immediate shock - December** | -2.43 | -21.56 - 16.69 | 0.80 | 5.76 | 8.10 - 19.62 | 0.41 | 7.55 | -17.47 - 32.56 | 0.55 | 1.22 | -17.26 - 19.71 | 0.90 | 35.05 | 17.68 - 52.42 | < 0.001 |
|  | **Post-event trend - December** | -0.03 | -0.13 - 0.08 | 0.61 | -0.07 | -0.14 - -0.01 | 0.03 | 0.01 | -0.12 - 0.13 | 0.90 | 0.10 | 0.002 - 0.20 | 0.05 | -0.18 | -0.27 - -0.08 | < 0.01 |

Note: Each model includes seven-day moving averages of reported COVID-19 cases and deaths as covariates.

Figure S7. Interrupted time-series regression analysis for the relative search volumes of online doctor, online health, telehealth, telemedicine, and health app before and after the announcement of the COVID-19 pandemic and the announcement of the first COVID-19 vaccines.


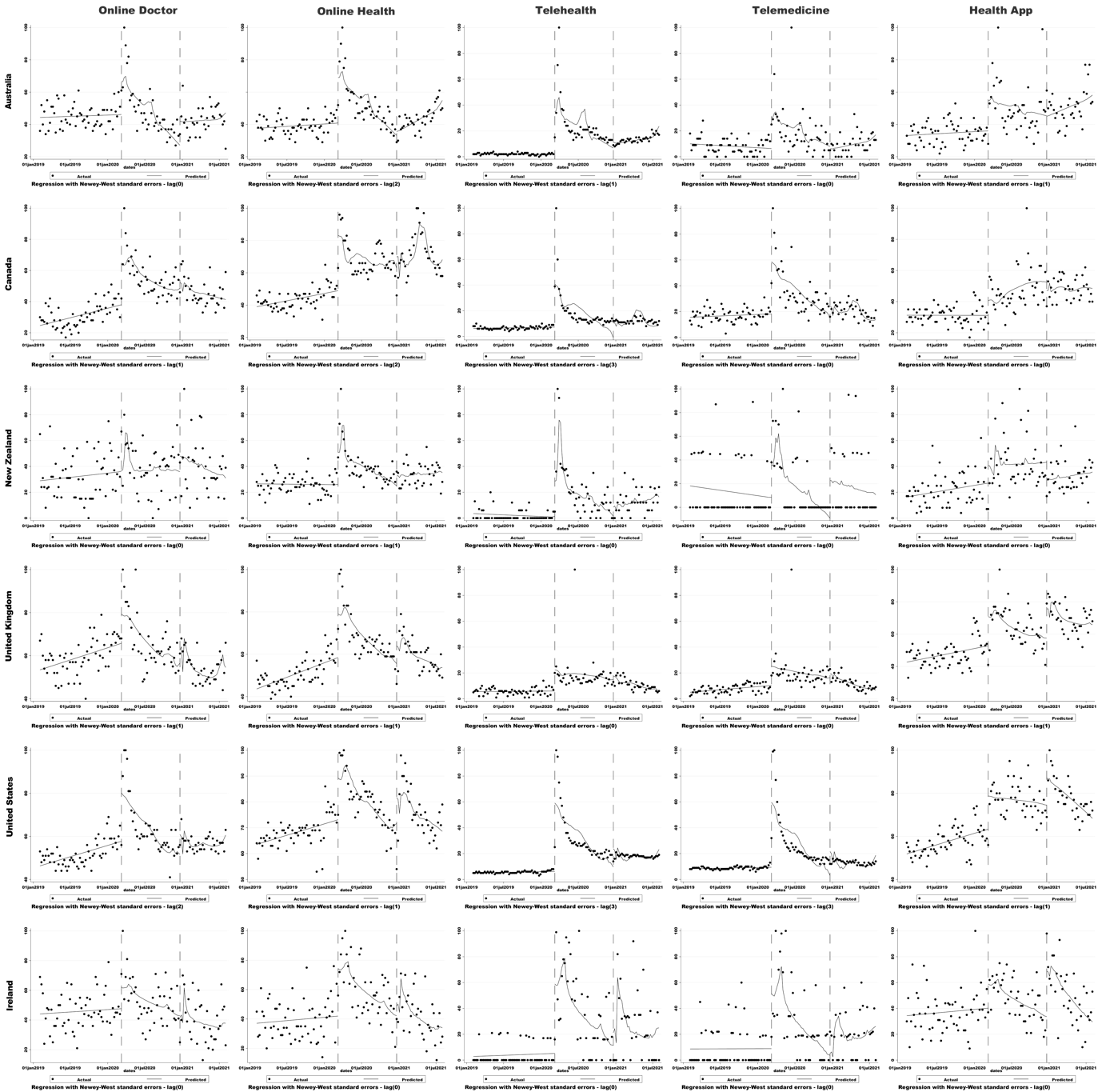


Note: the models include seven-day moving averages of reported COVID-19 cases and deaths as covariates. The first interruption occurs at 11 March 2020 and the second at 20 December 2020.

# Table S3. Results of the sequential estimation of break dates.

| **Bai & Perron Critical Values** | | | |
| --- | --- | --- | --- |
| Test statistic | 1% Critical value | 5% Critical value | 10% Critical value |
| 70.82 | 12.29 | 8.58 | 7.04 |

Estimated break points: 08 March 2020 – 14 March 2020

Trimming: 0.15

Figure S8. Interrupted time-series regression analysis for the relative search volumes of online doctor, online health, telehealth, telemedicine, and health app in the time period February 2017 to August 2019.


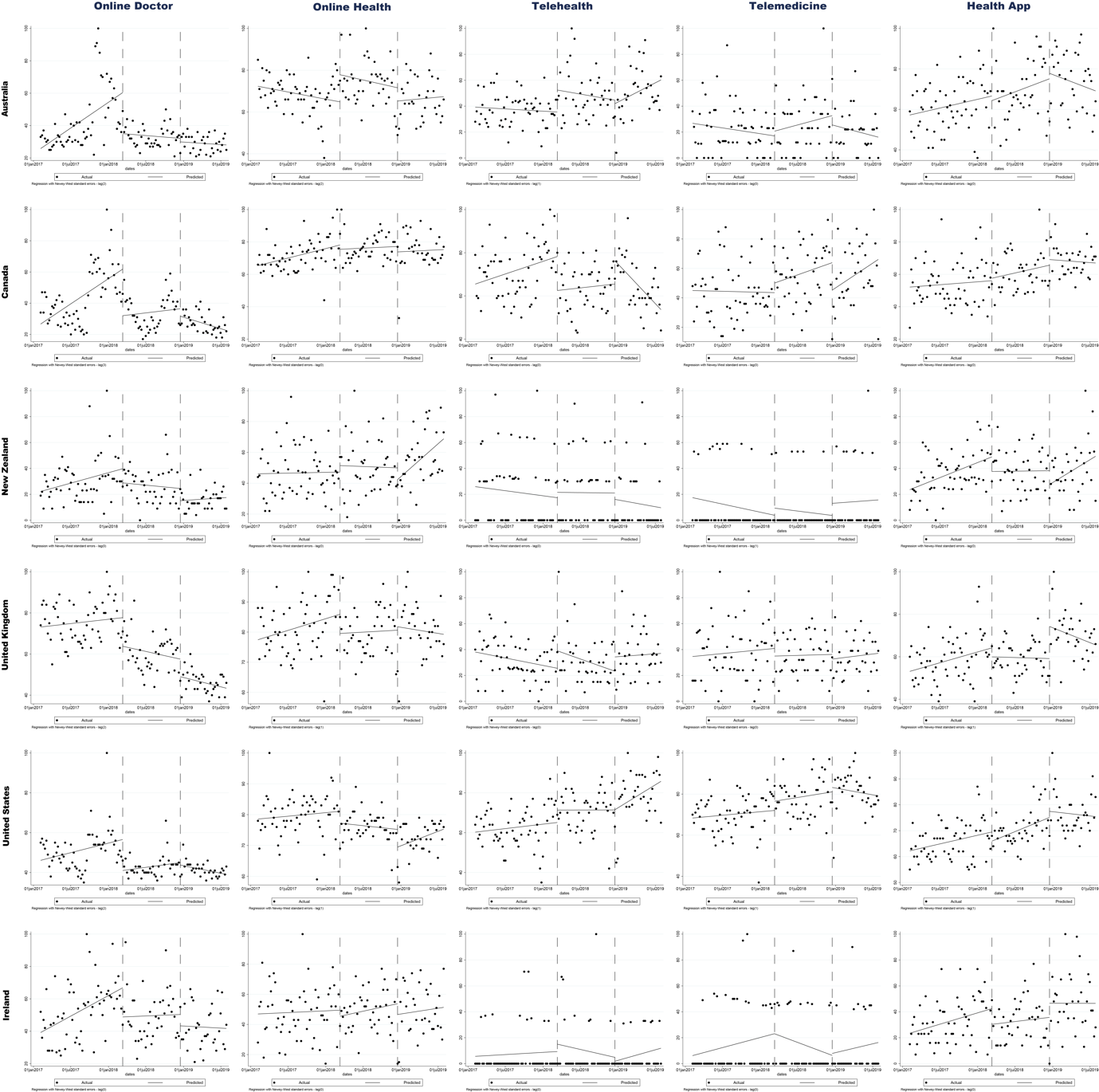


Note: The first interruption occurs at 8 March 2018 and the second at 16 December 2018.

# Table S4. Country-specific regression estimates for the relative search volumes of *online doctor*, *online health*, *telehealth*, *telemedicine*, and *health app* in the placebo period of 2017-2019.

|  |  | **Online Doctor** | | | **Online Health** | | | **Telehealth** | | | **Telemedicine** | | | **Health App** | | |
| --- | --- | --- | --- | --- | --- | --- | --- | --- | --- | --- | --- | --- | --- | --- | --- | --- |
|  |  | **Coefficient** | **95% CI** | **p-value** | **Coefficient** | **95% CI** | **p-value** | **Coefficient** | **95% CI** | **p-value** | **Coefficient** | **95% CI** | **p-value** | **Coefficient** | **95% CI** | **p-value** |
| **Australia** | **Intercept** | 25.80 | 20.05 - 31.55 | < 0,001 | 72.32 | 68.39 - 76.26 | < 0.001 | 39.25 | 32.72 - 45.78 | < 0.001 | 26.75 | 16.99 - 36.51 | < 0.001 | 57.23 | 51.51 - 62.94 | < 0.001 |
|  | **Pre-event trend - March** | 0,09 | 0,04 - 0,13 | < 0,001 | -0.02 | -0.05 - 0.01 | 0.21 | -0.01 | -0.03 - 0.02 | 0.46 | -0.02 | -0.06 - 0.01 | 0.18 | 0.02 | -0.004 - 0.05 | 0.09 |
|  | **Immediate shock - March** | -25.36 | -40.89 - -9.83 | < 0,001 | 12.99 | 2.19 - 23.80 | 0.02 | 16.94 | 1.82 - 32.06 | 0.03 | 3.66 | -10.19 - 17.53 | 0.60 | -2.28 | -14.91 - 10.35 | 0.72 |
|  | **Post-event trend - March** | -0.01 | -0.04 - 0.02 | 0.50 | -0.02 | -0.06 - 0.02 | 0.30 | -0.03 | -0.10 - 0.04 | 0.43 | 0.04 | -0.04 - 0.13 | 0.33 | 0.04 | -0.03 - 0.11 | 0.26 |
|  | **Immediate shock - December** | -2.16 | -8.15 - 3.84 | 0.48 | -6.36 | -15.76 - 3.04 | 0.18 | -2.74 | -18.29 - 12.81 | 0.73 | -7.19 | -27.41 - 13.02 | 0.48 | 2.70 | -11.34 - 16.74 | 0.70 |
|  | **Post-event trend - December** | -0.01 | -0.03 - 0.01 | 0.42 | 0.01 | -0.06 - 0.07 | 0.78 | 0.08 | -0.02 - 0.18 | 0.11 | -0.04 | -0.13 - 0.05 | 0.36 | -0.04 | -0.11 - 0.03 | 0.28 |
| **Canada** | **Intercept** | 26.66 | 17.27 - 36.05 | < 0,001 | 65.39 | 61.24 - 69.54 | < 0.001 | 65.49 | 59.82 - 71.16 | < 0.001 | 45.01 | 35.83 - 54.19 | < 0.001 | 52.14 | 45.39 - 58.89 | < 0.001 |
|  | **Pre-event trend - March** | 0.09 | 0.04 - 0.13 | < 0,001 | 0.03 | 0.01 - 0.06 | < 0.01 | 0.03 | 0.01 - 0.06 | 0.02 | -0.004 | -0.04 - 0.03 | 0.84 | 0.01 | -0.02 - 0.04 | 0.54 |
|  | **Immediate shock - March** | -29.84 | -47.53 - -12.15 | < 0,01 | -2.67 | -11.89 - 6.56 | 0.57 | -15.72 | -24.95 - -6.49 | < 0.01 | 6.54 | -5.67 - 18.76 | 0.29 | 1.54 | -8.74 - 11.82 | 0.77 |
|  | **Post-event trend - March** | 0.02 | -0,05 - 0.08 | 0.65 | 0.01 | -0.03 - 0.05 | 0.76 | 0.01 | -0.03 - 0.05 | 0.59 | 0.05 | -0.01 - 0.11 | 0.10 | 0.03 | -0.02 - 0.08 | 0.23 |
|  | **Immediate shock - December** | -4.78 | -15.80 - 6.24 | 0.39 | -3.39 | -15.43 - 8.65 | 0.58 | 10.82 | 2.62 - 19.01 | 0.01 | -18.67 | -36.89 - -0.45 | 0.05 | 3.48 | -7.55 - 14.51 | 0.53 |
|  | **Post-event trend - December** | -0.04 | -0.06 - -0.02 | < 0,001 | 0.01 | -0.06 - 0.08 | 0.85 | -0.10 | -0.14 - -0.06 | < 0.001 | 0.09 | -0.04 - 0.22 | 0.16 | -0.01 | -0.06 - 0.04 | 0.72 |
| **New Zealand** | **Intercept** | 22.21 | 16.59 - 27.84 | < 0,001 | 46.13 | 38.11 - 54.16 | < 0.001 | 25.93 | 12.29 - 39.56 | < 0.001 | 17.43 | 7.29 - 27.58 | < 0.01 | 23.62 | 16.03 - 31.20 | < 0.001 |
|  | **Pre-event trend - March** | 0.04 | 0.01 - 0.08 | 0.01 | 0.003 | -0.03 - 0.03 | 0.86 | -0.02 | -0.08 - 0.04 | 0.48 | -0.03 | -0.08 - 0.01 | 0.10 | 0.06 | 0.03 - 0.10 | < 0.001 |
|  | **Immediate shock - March** | -10.91 | -22.85 - 1.03 | 0.07 | 4.20 | -9.45 - 17.86 | 0.54 | 4.12 | -16.79 - 25.04 | 0.70 | 5.51 | -8.21 - 19.22 | 0.43 | -11.31 | -24.13 - 1.52 | 0.08 |
|  | **Post-event trend - March** | -0.02 | -0.06 - 0.03 | 0.53 | -0.01 | -0.07 - 0.06 | 0.87 | -0.002 | -0.09 - 0.09 | 0.97 | -0.02 | -0.08 - 0.04 | 0.51 | 0.002 | -0.06 - 0.06 | 0.94 |
|  | **Immediate shock - December** | -9.43 | -20.46 - 1.59 | 0.09 | -7.58 | -19.90 - 4.73 | 0.23 | -4.91 | -24.38 - 14.57 | 0.62 | 9.32 | -7.22 - 25.86 | 0.27 | -10.68 | -23.79 - 2.43 | 0.11 |
|  | **Post-event trend - December** | 0.01 | -0.03 - 0.05 | 0.59 | 0.12 | 0.04 - 0.19 | < 0.01 | -0.03 | -0.12 - 0.06 | 0.52 | 0.01 | -0.10 - 0.13 | 0.84 | 0.10 | -0.01 - 0.20 | 0.06 |
| **United Kingdom** | **Intercept** | 73.17 | 66.92 - 79.42 | < 0,001 | 77.46 | 73.25 - 81.67 | < 0.001 | 38.10 | 29.24 - 46.96 | < 0.001 | 34.63 | 23.96 - 45.31 | < 0.001 | 53.13 | 48.06 - 58.19 | < 0.001 |
|  | **Pre-event trend - March** | 0.01 | -0.02 - 0.04 | 0.45 | 0.02 | -0.002 - 0.04 | 0.07 | -0.03 | -0.07 - 0.01 | 0.10 | 0.02 | -0.03 - 0.06 | 0.51 | 0.03 | -0.001 - 0.06 | 0.06 |
|  | **Immediate shock - March** | -13.92 | -23.94 - -3.91 | < 0,01 | -6.37 | -13.78 - 1.03 | 0.09 | 13.44 | -3.35 - 30.24 | 0.12 | -5.91 | -20.45 - 8.64 | 0.42 | -4.23 | -13.34 - 4.88 | 0.36 |
|  | **Post-event trend - March** | -0.02 | -0.06 - 0.01 | 0.19 | 0.004 | -0.03 - 0.03 | 0.80 | -0.06 | -0.14 - 0.03 | 0.18 | 0.01 | -0.06 - 0.07 | 0.88 | -0.003 | -0.03 - 0.03 | 0.86 |
|  | **Immediate shock - December** | -8.54 | -14.31 - -2.77 | < 0,01 | 1.14 | -9.88 - 12.16 | 0.84 | 11.02 | -5.87 - 27.92 | 0.20 | -3.60 | -17.65 - 10.45 | 0.61 | 15.11 | 3.14 - 27.07 | 0.01 |
|  | **Post-event trend - December** | -0.02 | -0.05 - 0.002 | 0.07 | -0.01 | -0.08 - 0.05 | 0.73 | 0.01 | -0.08 - 0.10 | 0.79 | 0.02 | -0.05 - 0.09 | 0.59 | -0.04 | -0.11 - 0.03 | 0.28 |
| **United States** | **Intercept** | 46.13 | 40.72 - 51.54 | < 0,001 | 78.58 | 74.39 - 82.76 | < 0.001 | 60.27 | 55.14 - 65.39 | < 0.001 | 68.36 | 63.63 - 73.09 | < 0.001 | 62.24 | 59.27 - 65.20 | < 0.001 |
|  | **Pre-event trend - March** | 0.03 | -0.002 - 0.05 | 0.07 | 0.01 | -0.01 - 0.02 | 0.50 | 0.01 | -0.02 - 0.04 | 0.42 | 0.01 | -0.02 - 0.03 | 0.47 | 0.02 | 0.0002 - 0.04 | 0.05 |
|  | **Immediate shock - March** | -15.57 | -23.72 - -7.42 | < 0,001 | -3.84 | -8.94 - 1.26 | 0.14 | 6.22 | -4.14 - 16.59 | 0.24 | 4.49 | -4.20 - 13.18 | 0.31 | -3.53 | -9.48 - 2.41 | 0.24 |
|  | **Post-event trend - March** | 0.01 | -0.01 - 0.04 | 0.22 | -0.01 | -0.03 - 0.01 | 0.49 | 0.0003 | -0.04 - 0.05 | 0.99 | 0.02 | -0.02 - 0.05 | 0.38 | 0.03 | 0.01 - 0.05 | < 0.01 |
|  | **Immediate shock - December** | -1.23 | -6.68 - 4.22 | 0.66 | -5.72 | -11.49 - 0.05 | 0.05 | 0.10 | -14.31 - 14.50 | 0.99 | 2.16 | -8.79 - 13.10 | 0.70 | 2.36 | -6.46 - 11.19 | 0.60 |
|  | **Post-event trend - December** | -0.02 | -0.04 - -0.002 | 0.03 | 0.03 | -0.01 - 0.06 | 0.10 | 0.06 | -0.02 - 0.15 | 0.14 | -0.02 | -0.08 - 0.04 | 0.55 | -0.01 | -0.06 - 0.05 | 0.75 |
| **Ireland** | **Intercept** | 39.22 | 31.73 - 46.72 | < 0,001 | 46.97 | 37.63 - 56.31 | < 0.001 | 5.64 | -1.36 - 12.63 | 0.11 | 6.19 | -3.00 - 15.38 | 0.19 | 23.47 | 16.46 - 30.49 | < 0.001 |
|  | **Pre-event trend - March** | 0.07 | 0.04 - 0.10 | < 0,001 | 0.01 | -0.03 - 0.05 | 0.76 | 0.01 | -0.02 - 0.04 | 0.58 | 0.04 | -0.01 - 0.09 | 0.08 | 0.05 | 0.01 - 0.08 | 0.01 |
|  | **Immediate shock - March** | -18.01 | -31.32 - -4.69 | < 0,01 | -4.03 | -15.46 - 7.40 | 0.49 | 5.69 | -11.82 - 23.19 | 0.52 | -0.07 | -20.92 - 20.78 | 0.99 | -12.19 | -24.87 - 0.49 | 0.06 |
|  | **Post-event trend - March** | 0.01 | -0.06 - 0.07 | 0.87 | 0.03 | -0.02 - 0.08 | 0.22 | 0.04 | -0.13 - 0.05 | 0.42 | -0.06 | -0.15 - 0.03 | 0.20 | 0.02 | -0.04 - 0.08 | 0.51 |
|  | **Immediate shock - December** | -7.00 | -19.79 - 5.78 | 0.28 | -7.16 | -23.78 - 9.46 | 0.40 | -3.01 | -18.29 - 12.27 | 0.70 | 1.50 | -17.08 - 20.07 | 0.87 | 10.93 | -8.09 - 29.95 | 0.26 |
|  | **Post-event trend - December** | -0.01 | -0.07 - 0.06 | 0.83 | 0.02 | -0.08 - 0.12 | 0.68 | 0.04 | -0.02 - 0.11 | 0.17 | 0.04 | -0.06 - 0.13 | 0.42 | -0.001 | -0.11 - 0.11 | 0.99 |
